# Crystal structure of 3-methyl-1-phenyl-6-propyl­amino-1*H*-pyrazolo[3,4-*b*]pyridine-5-carbo­nitrile

**DOI:** 10.1107/S2056989015017004

**Published:** 2015-09-17

**Authors:** Jerry P. Jasinski, Mehmet Akkurt, Shaaban K. Mohamed, Hajjaj H. M. Abdu-Allah, Mustafa R. Albayati

**Affiliations:** a Department of Chemistry, Keene State College, 229 Main Street, Keene, NH 03435-2001, USA; b Department of Physics, Faculty of Sciences, Erciyes University, 38039 Kayseri, Turkey; c Chemistry and Environmental Division, Manchester Metropolitan University, Manchester M1 5GD, England; d Chemistry Department, Faculty of Science, Minia University, 61519 El-Minia, Egypt; e Department of Pharmaceutical Organic Chemistry, Faculty of Pharmacy, Assiut University, 71526 Assiut, Egypt; f Kirkuk University, College of Science, Department of Chemistry, Kirkuk, Iraq

**Keywords:** crystal structure, pyrazolo­[3,4-*b*]pyridine, amination, nucleophilic substitution

## Abstract

In the title compound, C_17_H_17_N_5_, the dihedral angle between the 1*H*-pyrazolo­[3,4-*b*]pyridine ring system (r.m.s. deviation = 0.001 Å) and the attached phenyl group is 2.56 (6)°. The propyl­amino side chain has a contorted conformation [C_ar_—N—C—C = −77.97 (16)° and N—C—C—C = −57.37 (17)°]. An intra­molecular C—H⋯N inter­action closes an *S*(6) ring. In the crystal, inversion dimers linked by pairs of N—H⋯N hydrogen bonds generate *R*_2_^2^(12) loops. Aromatic π–π stacking inter­actions [centroid–centroid distance = 3.5726 (8) Å] are also observed.

## Related literature   

For the chemistry of pyrazolo­[3,4-*b*]pyridines, see: Häufel & Breitmaier (1974 ▸); El-emary (2007 ▸); Dodiya *et al.* (2013 ▸). For a similar structure, see: Wang & Zhu (2006 ▸).
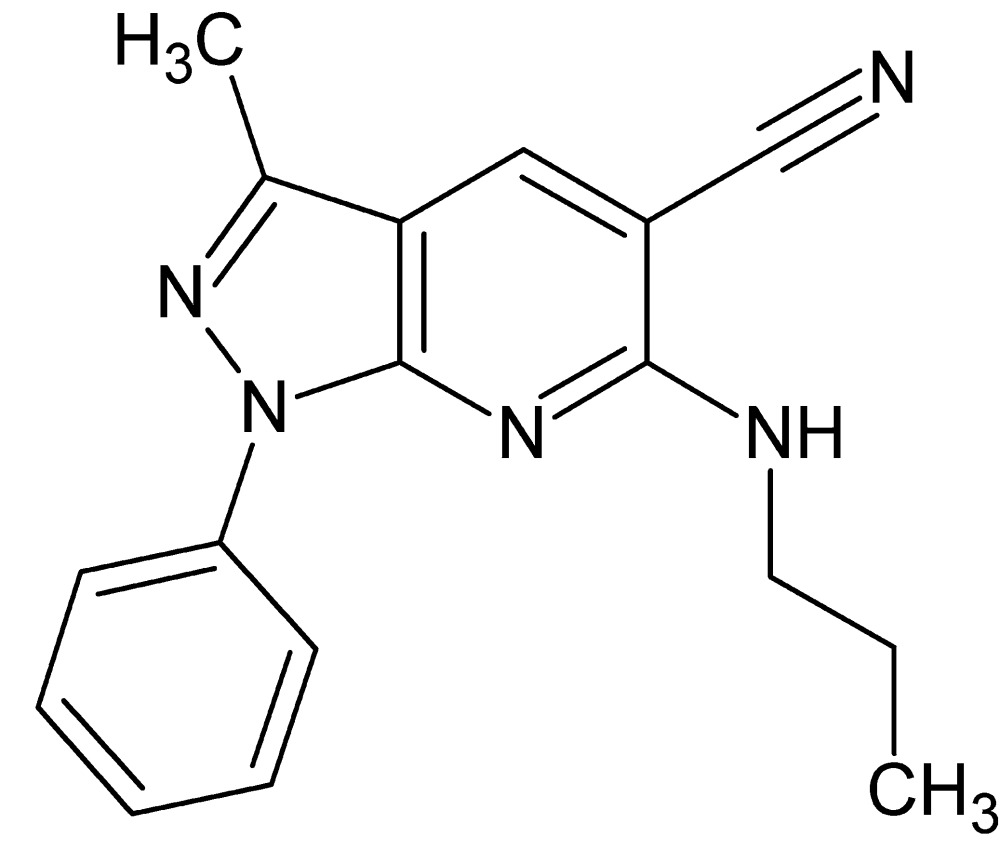


## Experimental   

### Crystal data  

C_17_H_17_N_5_
*M* *_r_* = 291.36
Monoclinic, 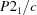

*a* = 5.1450 (2) Å
*b* = 15.1359 (7) Å
*c* = 19.5828 (9) Å
β = 96.547 (4)°
*V* = 1515.05 (12) Å^3^
*Z* = 4
Mo *K*α radiation
μ = 0.08 mm^−1^
*T* = 296 K
0.44 × 0.22 × 0.18 mm

### Data collection  

Agilent Xcalibur Eos Gemini diffractometer
Absorption correction: multi-scan (*CrysAlis PRO*; Agilent, 2014 ▸) *T*_min_ = 0.795, *T*_max_ = 1.000
10807 measured reflections
5031 independent reflections
3834 reflections with *I* > 2σ(*I*)
*R*_int_ = 0.025

### Refinement  

*R*[*F* ^2^ > 2σ(*F* ^2^)] = 0.055
*wR*(*F* ^2^) = 0.143
*S* = 1.03
5031 reflections
205 parameters
H atoms treated by a mixture of independent and constrained refinement
Δρ_max_ = 0.39 e Å^−3^
Δρ_min_ = −0.19 e Å^−3^

### 

Data collection: *CrysAlis PRO* (Agilent, 2014 ▸); cell refinement: *CrysAlis PRO*; data reduction: *CrysAlis PRO*; program(s) used to solve structure: *SHELXS2014* (Sheldrick, 2008 ▸); program(s) used to refine structure: *SHELXL2014* (Sheldrick, 2015 ▸); molecular graphics: *ORTEP-3 for Windows* (Farrugia, 2012 ▸); software used to prepare material for publication: *PLATON* (Spek, 2009 ▸).

## Supplementary Material

Crystal structure: contains datablock(s) global, I. DOI: 10.1107/S2056989015017004/hb7504sup1.cif

Structure factors: contains datablock(s) I. DOI: 10.1107/S2056989015017004/hb7504Isup2.hkl

Supporting information file. DOI: 10.1107/S2056989015017004/hb7504Isup3.cml

**Fig. 1.**
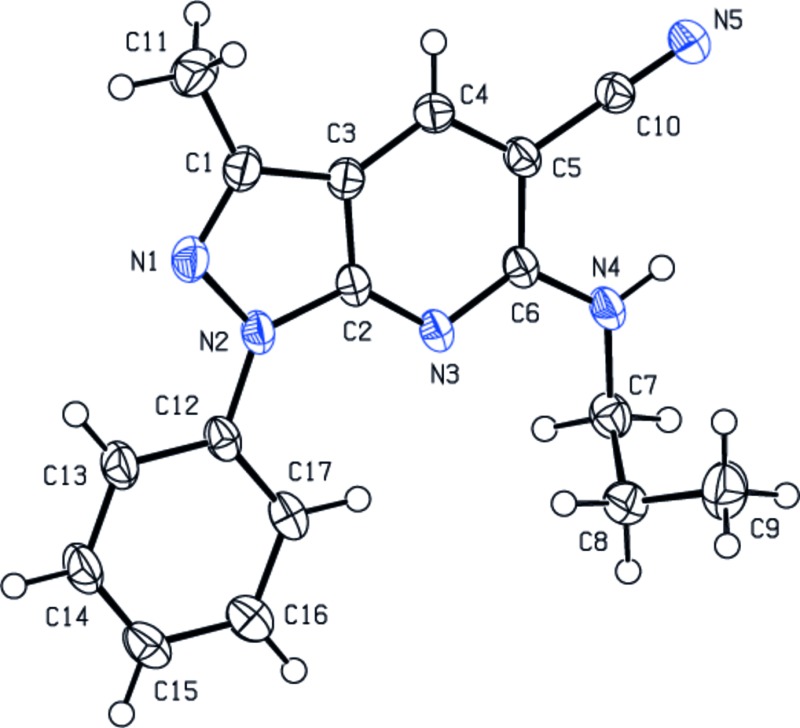
View of the title compound with displacement ellipsoids for non-H atoms drawn at the 50% probability level.

**Fig. 2.**
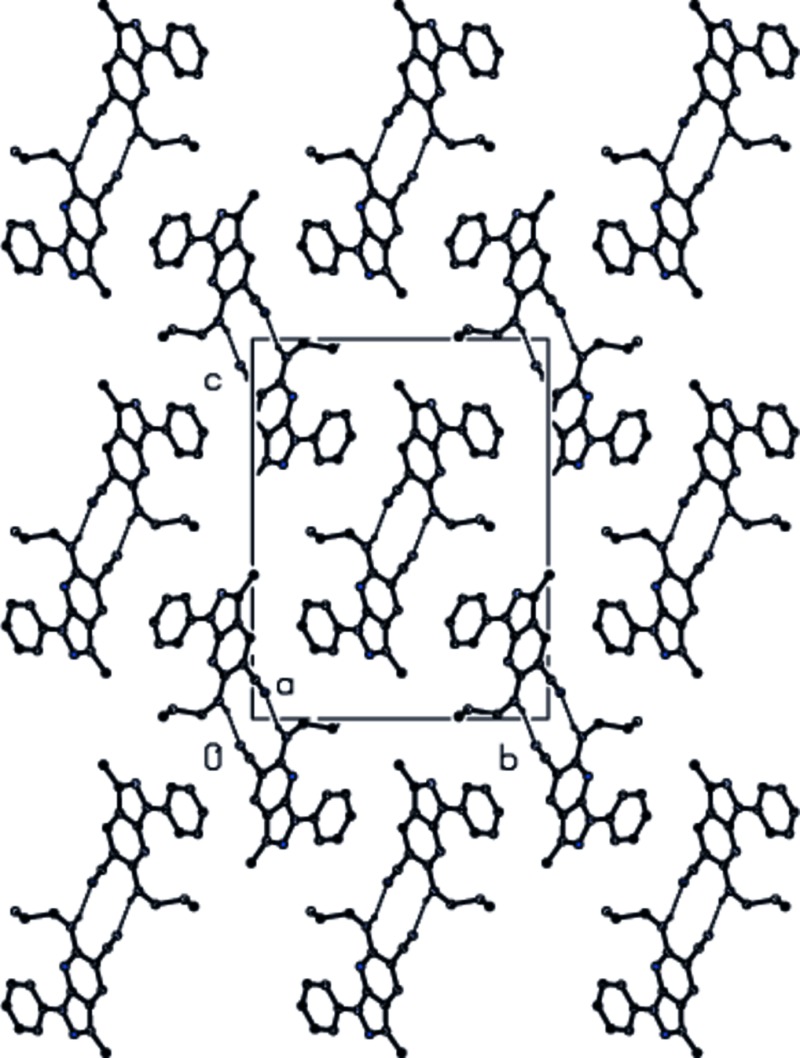
A part of N—H···O dimers viewed down b axis. H atoms not involved in H bonding are omitted for clarity.

. DOI: 10.1107/S2056989015017004/hb7504fig1.tif
View of the title compound with displacement ellipsoids for non-H atoms drawn at the 50% probability level.

b . DOI: 10.1107/S2056989015017004/hb7504fig2.tif
A part of N—H⋯O dimers viewed down *b* axis. H atoms not involved in H bonding are omitted for clarity.

CCDC reference: 1423566

Additional supporting information:  crystallographic information; 3D view; checkCIF report

## Figures and Tables

**Table 1:** Hydrogen-bond geometry (, )

*D*H*A*	*D*H	H*A*	*D* *A*	*D*H*A*
C17H17N3	0.93	2.33	2.9823(18)	127
N4H4*N*N5^i^	0.87(2)	2.20(2)	3.0326(17)	160.1(18)

Symmetry code: (i) 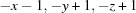.

## supplementary crystallographic information

### S1. Comment

Pyrazolo[3,4-*b*]pyridines (Häufel & Breitmaier, 1974; El-Emary *et al.*, 2007; Dodiya *et al.*, 2013) are attractive targets in organic synthesis and are being extensively investigated because of their wide range of biological activities. We are interested in the synthesis of 6-amino derivatives of 3-methyl-1-phenyl-6-chloro-1*H*-pyrazolo[3,4-*b*]pyridine-5-carbonitrile for potential biological interest.

The molecular structure of the title compound is shown in Fig. 1. The 1*H*-pyrazolo[3,4-*b*]pyridine ring system (N1—N3/C1—C6) is essentially planar [r.m.s. deviation = 0.001 Å]. It forms a dihedral angle of 2.56 (6) ° with the attached phenyl ring (C12—C17). All bond lengths and bond angles in the title compound are comparable with those of a similar structure previously published (Wang & Zhu, 2006).

In the crystal, pairs of N—H···O hydrogen bonds connect the molecules to each other, forming centrosymmetric dimers with *R*^2^_2_(12) motifs (Table 1, Fig. 2). π-π stacking interactions between the dimers [*Cg*1···*Cg*3(-1 + *x*, *y*, *z*) = 3.5726 (8) Å] between the centroids of the phenyl and pyrazole rings of the molecules] are also observed.

### S2. Experimental

A mixture of 3-methyl-1-phenyl-6-chloro-1*H*-pyrazolo[3,4-*b*]pyridine-5-carbonitrile (0.268 g, 1 mmol) and propyl amine (1.2 ml, 12 mmol) was heated to 323 K overnight with constant stirring. The reaction mixture was cooled to room temperature and taken up in dichloromethane, washed with 5% aq. NaHCO_3_, water and then with brine. The organic layer was dried over anhydrous MgSO_4_ and the solvent was removed under reduced pressure. The crude product was recrystallized from aqueous ethanol to give the title compound as colourless prisms (0.2 g, 69% yield); *R*_f_ = 0.25 (hexane:ethyl acetate, 4:1).

### S3. Refinement

The hydrogen atoms attached to carbon atoms were positioned geometrically and constrained to ride on their parent atoms, with carbon hydrogen bond distances of 0.93 - 0.97 Å. *U*_iso_(H) values were set to a multiple of *U*_eq_(C) with 1.5 for CH_3_ and 1.2 for CH and CH_2_, respectively. Reflection (-1 1 1) was affected by the beam stop and was omitted from the refinement. The H atom of the NH group were found from a difference Fourier map and refinned freely.

### Crystal data

**Table:**

C_17_H_17_N_5_	*F*(000) = 616
*M**_r_* = 291.36	*D*_x_ = 1.277 Mg m^−^^3^
Monoclinic, *P*2_1_/*c*	Mo *K*α radiation, λ = 0.71073 Å
Hall symbol: -P 2ybc	Cell parameters from 2809 reflections
*a* = 5.1450 (2) Å	θ = 4.1–32.4°
*b* = 15.1359 (7) Å	µ = 0.08 mm^−^^1^
*c* = 19.5828 (9) Å	*T* = 296 K
β = 96.547 (4)°	Prism, colourless
*V* = 1515.05 (12) Å^3^	0.44 × 0.22 × 0.18 mm
*Z* = 4	

### Data collection

**Table:**

Agilent Xcalibur Eos Gemini diffractometer	5031 independent reflections
Radiation source: Enhance (Mo) X-ray Source	3834 reflections with *I* > 2σ(*I*)
Graphite monochromator	*R*_int_ = 0.025
Detector resolution: 16.0416 pixels mm^-1^	θ_max_ = 32.7°, θ_min_ = 3.4°
ω scans	*h* = −7→7
Absorption correction: multi-scan (*CrysAlis PRO*; Agilent, 2014)	*k* = −21→19
*T*_min_ = 0.795, *T*_max_ = 1.000	*l* = −29→17
10807 measured reflections	

### Refinement

**Table:**

Refinement on *F*^2^	0 restraints
Least-squares matrix: full	Hydrogen site location: mixed
*R*[*F*^2^ > 2σ(*F*^2^)] = 0.055	H atoms treated by a mixture of independent and constrained refinement
*wR*(*F*^2^) = 0.143	*w* = 1/[σ^2^(*F*_o_^2^) + (0.0575*P*)^2^ + 0.5443*P*] where *P* = (*F*_o_^2^ + 2*F*_c_^2^)/3
*S* = 1.03	(Δ/σ)_max_ < 0.001
5031 reflections	Δρ_max_ = 0.39 e Å^−^^3^
205 parameters	Δρ_min_ = −0.19 e Å^−^^3^

### Special details

**Table:**

Geometry. Bond distances, angles *etc*. have been calculated using the rounded fractional coordinates. All su's are estimated from the variances of the (full) variance-covariance matrix. The cell e.s.d.'s are taken into account in the estimation of distances, angles and torsion angles
Refinement. Refinement on *F*^2^ for ALL reflections except those flagged by the user for potential systematic errors. Weighted *R*-factors *wR* and all goodnesses of fit *S* are based on *F*^2^, conventional *R*-factors *R* are based on *F*, with *F* set to zero for negative *F*^2^. The observed criterion of *F*^2^ > σ(*F*^2^) is used only for calculating -*R*-factor-obs *etc*. and is not relevant to the choice of reflections for refinement. *R*-factors based on *F*^2^ are statistically about twice as large as those based on *F*, and *R*-factors based on ALL data will be even larger.

### Fractional atomic coordinates and isotropic or equivalent isotropic displacement parameters (Å^2^)

**Table:**

	*x*	*y*	*z*	*U*_iso_*/*U*_eq_	
N1	0.1143 (2)	0.39657 (8)	0.17034 (6)	0.0313 (3)	
N2	0.1880 (2)	0.35673 (7)	0.23372 (5)	0.0259 (3)	
N3	0.0577 (2)	0.36544 (7)	0.34854 (5)	0.0240 (3)	
N4	−0.1030 (2)	0.38566 (8)	0.45253 (6)	0.0290 (3)	
N5	−0.6220 (3)	0.54182 (8)	0.43046 (6)	0.0335 (3)	
C1	−0.0775 (3)	0.45129 (9)	0.17907 (7)	0.0307 (4)	
C2	0.0384 (2)	0.38812 (8)	0.28219 (6)	0.0235 (3)	
C3	−0.1361 (3)	0.44952 (8)	0.24825 (7)	0.0263 (3)	
C4	−0.3133 (3)	0.49088 (8)	0.28667 (7)	0.0275 (3)	
C5	−0.3023 (2)	0.46884 (8)	0.35533 (7)	0.0250 (3)	
C6	−0.1121 (2)	0.40572 (8)	0.38532 (6)	0.0238 (3)	
C7	0.0940 (3)	0.32792 (9)	0.48825 (7)	0.0305 (4)	
C8	0.0446 (3)	0.22989 (10)	0.47497 (8)	0.0337 (4)	
C9	−0.2167 (4)	0.19810 (12)	0.49344 (10)	0.0464 (5)	
C10	−0.4806 (3)	0.50939 (8)	0.39696 (7)	0.0264 (3)	
C11	−0.2089 (4)	0.50428 (11)	0.12099 (8)	0.0445 (5)	
C12	0.3868 (2)	0.29171 (8)	0.23836 (7)	0.0259 (3)	
C13	0.5115 (3)	0.27319 (9)	0.18042 (8)	0.0340 (4)	
C14	0.7117 (3)	0.21152 (10)	0.18464 (9)	0.0386 (4)	
C15	0.7886 (3)	0.16777 (9)	0.24520 (9)	0.0356 (4)	
C16	0.6611 (3)	0.18531 (10)	0.30210 (8)	0.0334 (4)	
C17	0.4609 (3)	0.24712 (9)	0.29935 (7)	0.0301 (4)	
H4	−0.43410	0.53170	0.26680	0.0330*	
H4N	−0.206 (4)	0.4127 (12)	0.4777 (10)	0.041 (5)*	
H7A	0.10150	0.33880	0.53730	0.0370*	
H7B	0.26340	0.34310	0.47430	0.0370*	
H8A	0.05460	0.21790	0.42670	0.0400*	
H8B	0.18220	0.19630	0.50130	0.0400*	
H9A	−0.22310	0.20520	0.54190	0.0700*	
H9B	−0.23880	0.13680	0.48150	0.0700*	
H9C	−0.35420	0.23200	0.46870	0.0700*	
H11A	−0.20430	0.56570	0.13330	0.0670*	
H11B	−0.38750	0.48550	0.11120	0.0670*	
H11C	−0.11980	0.49580	0.08100	0.0670*	
H13	0.46060	0.30210	0.13910	0.0410*	
H14	0.79530	0.19950	0.14600	0.0460*	
H15	0.92450	0.12700	0.24780	0.0430*	
H16	0.71030	0.15520	0.34290	0.0400*	
H17	0.37710	0.25850	0.33810	0.0360*	

### Atomic displacement parameters (Å^2^)

**Table:**

	*U*^11^	*U*^22^	*U*^33^	*U*^12^	*U*^13^	*U*^23^
N1	0.0380 (6)	0.0317 (6)	0.0259 (5)	0.0000 (5)	0.0115 (5)	0.0002 (4)
N2	0.0282 (5)	0.0285 (5)	0.0227 (5)	−0.0009 (4)	0.0100 (4)	−0.0025 (4)
N3	0.0231 (5)	0.0257 (5)	0.0243 (5)	−0.0018 (4)	0.0080 (4)	−0.0049 (4)
N4	0.0303 (6)	0.0340 (6)	0.0244 (5)	0.0061 (5)	0.0104 (4)	−0.0027 (4)
N5	0.0361 (6)	0.0338 (6)	0.0319 (6)	0.0063 (5)	0.0102 (5)	−0.0022 (5)
C1	0.0382 (7)	0.0286 (6)	0.0268 (6)	−0.0021 (5)	0.0102 (5)	0.0009 (5)
C2	0.0238 (5)	0.0235 (5)	0.0245 (6)	−0.0039 (4)	0.0080 (4)	−0.0044 (4)
C3	0.0312 (6)	0.0229 (5)	0.0260 (6)	−0.0025 (5)	0.0089 (5)	−0.0014 (4)
C4	0.0302 (6)	0.0222 (5)	0.0310 (6)	0.0000 (5)	0.0078 (5)	−0.0006 (5)
C5	0.0249 (6)	0.0221 (5)	0.0293 (6)	−0.0007 (4)	0.0085 (5)	−0.0039 (4)
C6	0.0231 (5)	0.0237 (5)	0.0256 (6)	−0.0025 (4)	0.0072 (4)	−0.0047 (4)
C7	0.0281 (6)	0.0374 (7)	0.0259 (6)	0.0016 (5)	0.0030 (5)	−0.0027 (5)
C8	0.0314 (7)	0.0361 (7)	0.0338 (7)	0.0048 (6)	0.0051 (5)	0.0022 (6)
C9	0.0460 (9)	0.0489 (9)	0.0465 (9)	−0.0075 (8)	0.0150 (8)	0.0028 (7)
C10	0.0280 (6)	0.0238 (5)	0.0282 (6)	−0.0001 (5)	0.0064 (5)	−0.0017 (5)
C11	0.0614 (11)	0.0417 (8)	0.0317 (8)	0.0091 (8)	0.0110 (7)	0.0074 (6)
C12	0.0242 (6)	0.0250 (6)	0.0298 (6)	−0.0049 (5)	0.0093 (5)	−0.0081 (5)
C13	0.0412 (8)	0.0314 (7)	0.0326 (7)	0.0002 (6)	0.0179 (6)	−0.0048 (5)
C14	0.0431 (8)	0.0327 (7)	0.0443 (8)	0.0004 (6)	0.0240 (7)	−0.0092 (6)
C15	0.0299 (7)	0.0293 (6)	0.0492 (9)	0.0002 (5)	0.0110 (6)	−0.0110 (6)
C16	0.0293 (7)	0.0345 (7)	0.0363 (7)	0.0008 (5)	0.0033 (5)	−0.0070 (6)
C17	0.0263 (6)	0.0359 (7)	0.0292 (6)	−0.0001 (5)	0.0083 (5)	−0.0079 (5)

### Geometric parameters (Å, º)

**Table:**

N1—N2	1.3930 (15)	C13—C14	1.386 (2)
N1—C1	1.3143 (18)	C14—C15	1.376 (2)
N2—C2	1.3732 (15)	C15—C16	1.382 (2)
N2—C12	1.4148 (15)	C16—C17	1.388 (2)
N3—C2	1.3366 (15)	C4—H4	0.9300
N3—C6	1.3406 (15)	C7—H7A	0.9700
N4—C6	1.3463 (17)	C7—H7B	0.9700
N4—C7	1.4556 (18)	C8—H8A	0.9700
N5—C10	1.144 (2)	C8—H8B	0.9700
C1—C3	1.421 (2)	C9—H9A	0.9600
C1—C11	1.490 (2)	C9—H9B	0.9600
C2—C3	1.4055 (18)	C9—H9C	0.9600
C3—C4	1.395 (2)	C11—H11A	0.9600
C4—C5	1.3802 (19)	C11—H11B	0.9600
N4—H4N	0.87 (2)	C11—H11C	0.9600
C5—C10	1.4330 (19)	C13—H13	0.9300
C5—C6	1.4437 (16)	C14—H14	0.9300
C7—C8	1.523 (2)	C15—H15	0.9300
C8—C9	1.510 (3)	C16—H16	0.9300
C12—C17	1.3869 (19)	C17—H17	0.9300
C12—C13	1.394 (2)		
			
N2—N1—C1	106.80 (11)	C3—C4—H4	121.00
N1—N2—C2	110.43 (10)	C5—C4—H4	121.00
N1—N2—C12	118.66 (10)	N4—C7—H7A	109.00
C2—N2—C12	130.87 (10)	N4—C7—H7B	109.00
C2—N3—C6	115.13 (10)	C8—C7—H7A	109.00
C6—N4—C7	123.36 (11)	C8—C7—H7B	109.00
N1—C1—C3	110.86 (12)	H7A—C7—H7B	108.00
N1—C1—C11	121.48 (13)	C7—C8—H8A	109.00
C3—C1—C11	127.65 (14)	C7—C8—H8B	109.00
N2—C2—N3	126.71 (11)	C9—C8—H8A	109.00
N2—C2—C3	106.27 (11)	C9—C8—H8B	109.00
N3—C2—C3	127.03 (11)	H8A—C8—H8B	108.00
C1—C3—C2	105.65 (12)	C8—C9—H9A	109.00
C1—C3—C4	136.75 (13)	C8—C9—H9B	109.00
C2—C3—C4	117.60 (12)	C8—C9—H9C	110.00
C3—C4—C5	117.40 (12)	H9A—C9—H9B	109.00
C7—N4—H4N	116.6 (13)	H9A—C9—H9C	109.00
C6—N4—H4N	119.6 (13)	H9B—C9—H9C	109.00
C4—C5—C10	119.53 (11)	C1—C11—H11A	109.00
C4—C5—C6	120.45 (11)	C1—C11—H11B	109.00
C6—C5—C10	120.02 (12)	C1—C11—H11C	109.00
N4—C6—C5	119.56 (11)	H11A—C11—H11B	109.00
N3—C6—C5	122.37 (11)	H11A—C11—H11C	110.00
N3—C6—N4	118.07 (11)	H11B—C11—H11C	109.00
N4—C7—C8	114.17 (12)	C12—C13—H13	120.00
C7—C8—C9	113.92 (13)	C14—C13—H13	120.00
N5—C10—C5	179.66 (15)	C13—C14—H14	120.00
N2—C12—C13	118.95 (12)	C15—C14—H14	120.00
C13—C12—C17	119.75 (12)	C14—C15—H15	120.00
N2—C12—C17	121.30 (12)	C16—C15—H15	120.00
C12—C13—C14	119.66 (14)	C15—C16—H16	119.00
C13—C14—C15	120.95 (15)	C17—C16—H16	119.00
C14—C15—C16	119.09 (14)	C12—C17—H17	120.00
C15—C16—C17	121.11 (14)	C16—C17—H17	120.00
C12—C17—C16	119.43 (13)		
			
C1—N1—N2—C2	−0.22 (14)	N1—C1—C3—C2	0.00 (16)
C1—N1—N2—C12	177.73 (11)	N3—C2—C3—C1	179.11 (12)
N2—N1—C1—C3	0.13 (15)	N2—C2—C3—C1	−0.15 (14)
N2—N1—C1—C11	−178.72 (13)	N3—C2—C3—C4	−1.3 (2)
C2—N2—C12—C17	0.2 (2)	N2—C2—C3—C4	179.47 (12)
N1—N2—C12—C17	−177.28 (12)	C1—C3—C4—C5	179.97 (16)
C12—N2—C2—N3	3.4 (2)	C2—C3—C4—C5	0.51 (19)
N1—N2—C2—C3	0.23 (13)	C3—C4—C5—C6	0.41 (18)
N1—N2—C2—N3	−179.03 (11)	C3—C4—C5—C10	−179.87 (12)
C2—N2—C12—C13	−179.14 (12)	C10—C5—C6—N3	179.49 (11)
C12—N2—C2—C3	−177.40 (12)	C4—C5—C6—N3	−0.79 (18)
N1—N2—C12—C13	3.40 (17)	C4—C5—C6—N4	179.43 (12)
C2—N3—C6—C5	0.14 (17)	C10—C5—C6—N4	−0.29 (17)
C6—N3—C2—C3	0.91 (18)	N4—C7—C8—C9	−57.37 (17)
C2—N3—C6—N4	179.92 (11)	N2—C12—C13—C14	178.16 (12)
C6—N3—C2—N2	−180.00 (12)	C17—C12—C13—C14	−1.2 (2)
C7—N4—C6—C5	−175.32 (11)	N2—C12—C17—C16	−178.48 (12)
C7—N4—C6—N3	4.89 (18)	C13—C12—C17—C16	0.8 (2)
C6—N4—C7—C8	−77.97 (16)	C12—C13—C14—C15	0.4 (2)
C11—C1—C3—C2	178.77 (14)	C13—C14—C15—C16	0.8 (2)
C11—C1—C3—C4	−0.7 (3)	C14—C15—C16—C17	−1.1 (2)
N1—C1—C3—C4	−179.50 (16)	C15—C16—C17—C12	0.3 (2)

### Hydrogen-bond geometry (Å, º)

**Table:**

*D*—H···*A*	*D*—H	H···*A*	*D*···*A*	*D*—H···*A*
C17—H17···N3	0.93	2.33	2.9823 (18)	127
N4—H4*N*···N5^i^	0.87 (2)	2.20 (2)	3.0326 (17)	160.1 (18)

Symmetry code: (i) −*x*−1, −*y*+1, −*z*+1.
