# Supplementary figures and images for: Crystal structure of 3-methyl-1-phenyl-6-propyl­amino-1H-pyrazolo[3,4-b]pyridine-5-carbo­nitrile
Source: Acta Crystallogr E Crystallogr Commun. 2015 Sep 17;71(Pt 10):o766–7. doi: 10.1107/S2056989015017004 (PMC4647426; doi:10.1107/S2056989015017004)

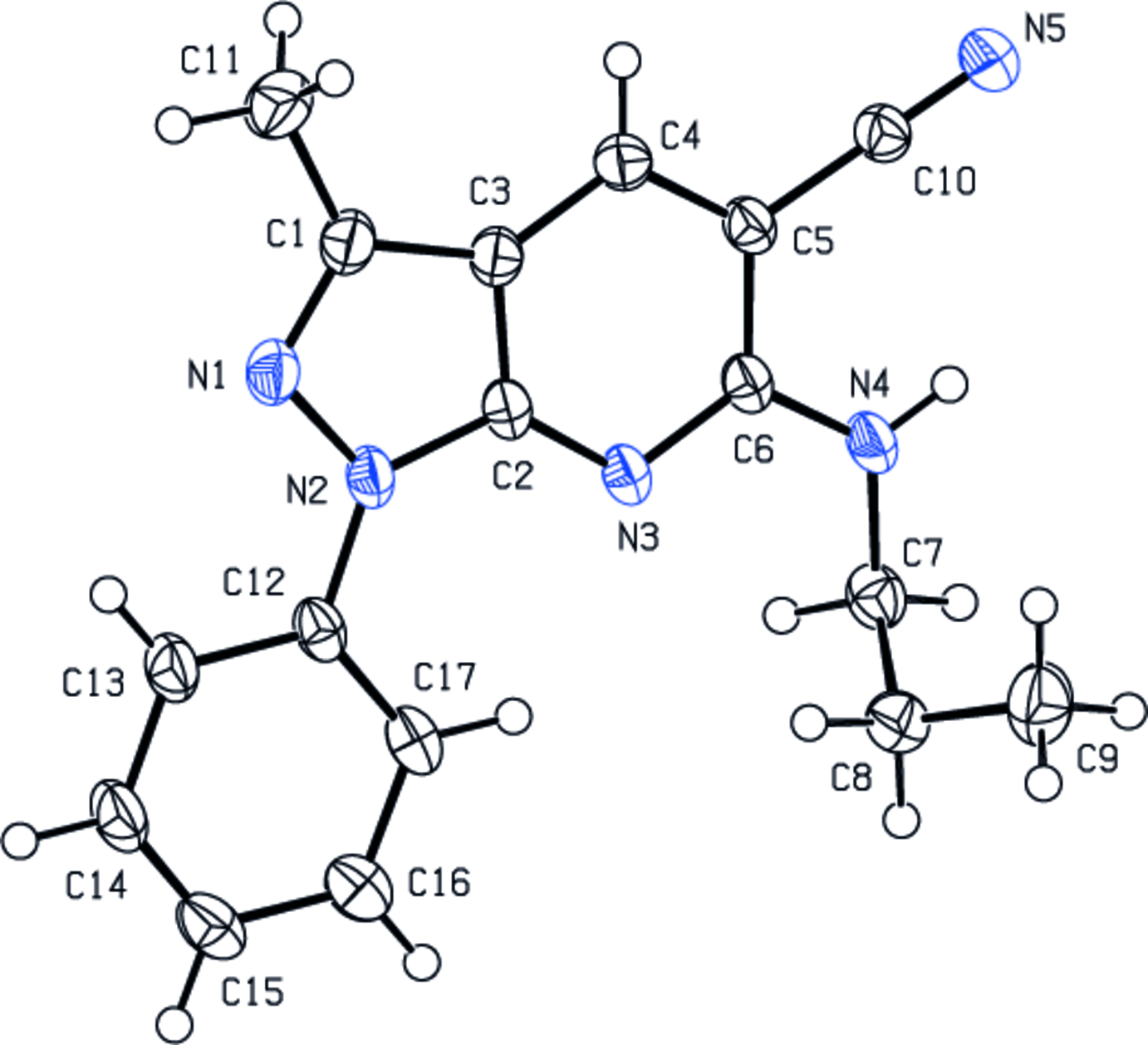

Supplement: Supplementary file 4 [file e-71-0o766-fig1.tif]

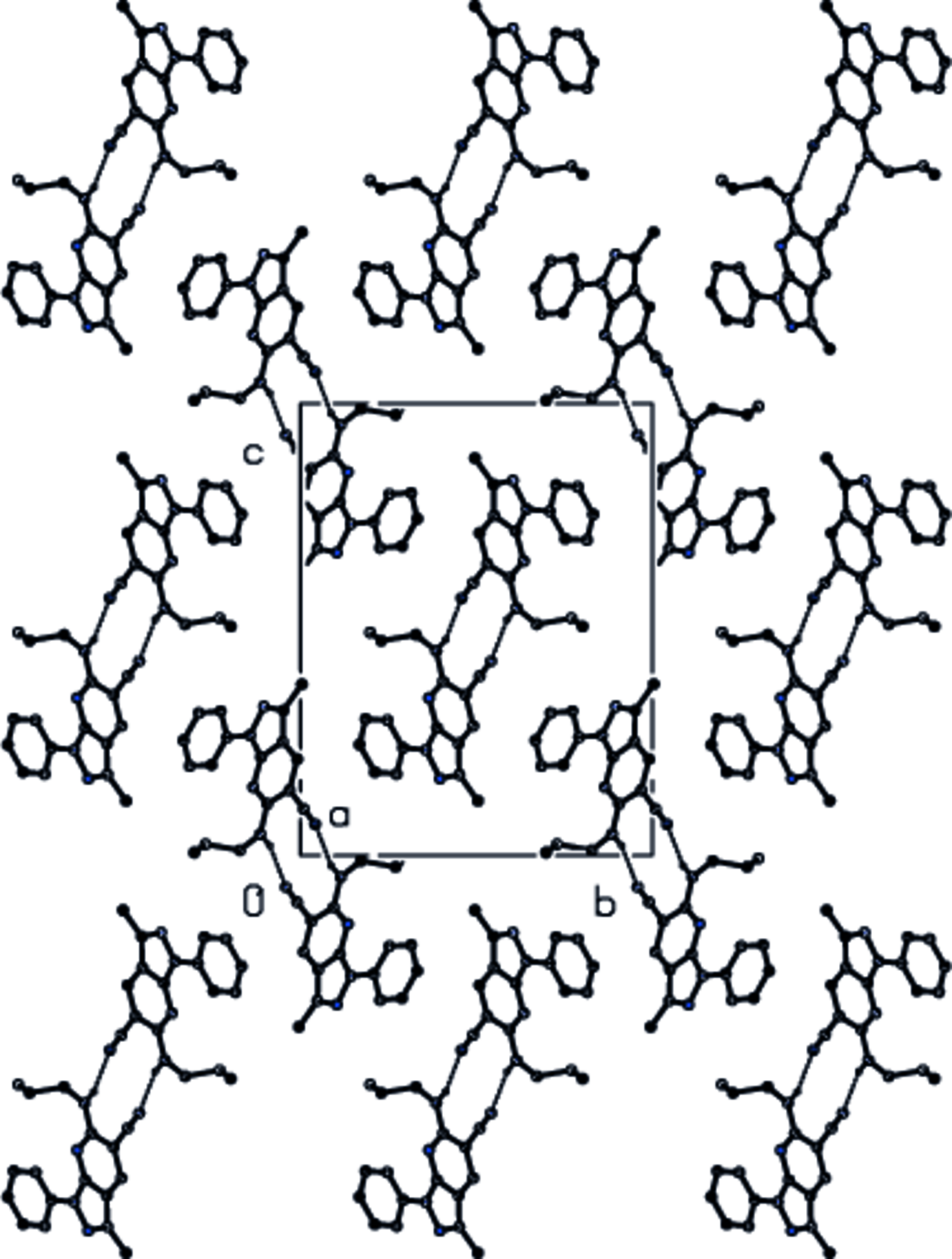

Supplement: Supplementary file 5 [file e-71-0o766-fig2.tif]
